# Commentary on ‘A new clinical severity score for the management of acute small bowel obstruction in predicting bowel ischemia: a cohort study’ [Int J Surg 2023; 109:1620–28]

**DOI:** 10.1097/JS9.0000000000000628

**Published:** 2023-08-17

**Authors:** Zengwu Yao, Yifei Zhang, Lixin Jiang

**Affiliations:** a Yantai Yuhuangding Hospital, Shandong University; b Department of Gastrointestinal Surgery, Yantai Yuhuangding Hospital affiliated with Qingdao University; c General Surgery, Yantai Yeda Hospital, Shandong Province, People’s Republic of China

*Dear Editor*,

We were interested in the article by Wassmer *et al*.^1^ titled ‘A new clinical severity score for the management of acute small bowel obstruction in predicting bowel ischemia: a cohort study.’ In their retrospective cohort study, Wassmer *et al*. developed and validated a practical clinical severity score designed to tailor the management of patients presenting with small bowel obstruction (SBO). Eight variables were found to be strongly associated with small bowel resection: age ≥70 years; first episode of SBO; no bowel movement for ≥3 days; abdominal guarding; C-reactive protein ≥50; and three abdominal computed tomography (CT) signs: small bowel transition point, lack of small bowel contrast enhancement, and presence of >500 ml of intra-abdominal fluid. The sensitivity and specificity of this score were 65% and 88%, respectively, and the area under the curve was 0.84 (95% confidence interval, 0.80–0.89). We thank the authors for providing valuable data in their study; however, we would like to provide a few comments.

First, although the sensitivity and specificity of this model were relatively high, some important factors may have been ignored. For example, leukocytosis (>10 000/mm^3^) did not exhibit a strong association; however, a previous study^2^ reported a strong correlation when the threshold was set to 12 000. Furthermore, as the authors mentioned, procalcitonin is increasingly used in the diagnosis of bacterial infections. It is a 116-amino acid peptide precursor of calcitonin that can help predict the failure of conservative management and the occurrence of bowel ischaemia in patients with SBO^3^. Moreover, lactic acid and creatinine are indicators that generally occur only when prolonged intestinal necrosis leads to severe septic shock. Therefore, increasing the threshold for leukocytosis and incorporating procalcitonin into the model may improve its sensitivity and specificity.

Second, although all common CT manifestations are listed in the article, other manifestations cannot be ignored. For example, Taghavifar *et al*.^4^ reported mesenteric oedema, a lack of small bowel faeces, bowel wall thickening, fat stranding in the mesentery, and an unusual course of the mesenteric vasculature (swirl sign) as novel CT findings that are associated with SBO^2,4^.

Third, another critical and simple method that is ignored in the study is judging according to the nature of the paracentesis fluid. In the cases where a large amount of peritoneal effusion is present, a small amount of peritoneal fluid is extracted using a syringe to observe its characteristics, such as shape, colour, and smell, which will assist in making an assessment. A light bloody, turbid fluid accompanied by a foul smell indicates the occurrence of intestinal ischaemia or necrosis in most cases. Additionally, ‘gradually worsening’ may be a more accurate term for the expression of peritonitis in the model. This is because once ischaemia or necrosis develops, the patient’s abdominal pain gradually increases and remains unrelieved.

In addition, the study should have considered another disease with very similar clinical symptoms and CT findings but a completely different treatment method–small bowel obstruction caused by oral warfarin^5^. Most patients had a history of oral warfarin and other anticoagulant drugs. Laboratory test results showed an abnormal prothrombin time and international normalised ratio. Oral vitamin K administration and conservative treatment helped relieve clinical signs and improve laboratory findings, and no surgical treatment was required.

Despite the above concerns, this is a valuable study that provides a new clinical severity score for managing acute SBO in predicting bowel ischaemia.

## Ethical approval

Not applicable.

## Consent

Not applicable.

## Sources of funding

Shandong University Cooperation Project (3460019005).

## Author contribution

Z.Y.: writing the paper; Z.Y., Y.Z., and L.J.: study concept or design; Z.Y. and Y.Z.: data collection and data analysis.

## Conflicts of interest disclosure

The authors declare that they have no conflicts of interest.

## Research registration unique identifying number (UIN)

Not applicable.

## Guarantor

Lixin Jiang.

## Data availability statement

Not applicable.

## Provenance and peer review

Commentary, internally reviewed.
